# Depolarization shift in the resting membrane potential of inferior colliculus neurons explains their hyperactivity induced by an acoustic trauma

**DOI:** 10.3389/fnins.2023.1258349

**Published:** 2023-09-05

**Authors:** Chun-Jen Hsiao, Alexander V. Galazyuk

**Affiliations:** Department of Anatomy and Neurobiology, Northeast Ohio Medical University, Rootstown, OH, United States

**Keywords:** *in-vivo* intracellular recording, sound exposure, tinnitus, hyperacusis, mice, inferior colliculus

## Abstract

**Introduction:**

Neuronal hyperactivity has been associated with many brain diseases. In the auditory system, hyperactivity has been linked to hyperacusis and tinnitus. Previous research demonstrated the development of hyperactivity in inferior colliculus (IC) neurons after sound overexposure, but the underlying mechanism of this hyperactivity remains unclear. The main goal of this study was to determine the mechanism of this hyperactivity.

**Methods:**

Experiments were performed on CBA/CaJ mice in a restrained, unanesthetized condition using intracellular recordings with sharp microelectrodes. Recordings were obtained from control (unexposed) and unilaterally sound overexposed groups of mice.

**Results:**

Our data suggest that sound exposure-induced hyperactivity was due to a depolarizing shift of the resting membrane potential (RMP) in the hyperactive neurons. The half width of action potentials in these neurons was also decreased after sound exposure. Surprisingly, we also found an RMP gradient in which neurons have more hyperpolarized RMPs with increasing depth in the IC. This gradient was altered in the overexposed animals.

## Introduction

Abnormal hyperactivity and hyperexcitability have been associated with numerous neurological disorders including epilepsy (Shevlyakov et al., 2023), Alzheimer’s Disease (Ali et al., 2022; Targa Dias Anastacio et al., 2022), Parkinson’s disease (Campanelli et al., 2022), retinal neurodegeneration (Telias et al., 2022), fragile X syndrome (Telias, 2019), tinnitus (Shore et al., 2016), and many others. In all cases, the uncontrolled firing of neurons in the central nervous system is connected to the disruption of existing circuits. The mechanisms underlying neuronal hyperactivity remain elusive. A careful assessment of the commonalities and disparities in the mechanisms for each pathology might uncover novel therapeutic targets and develop potential treatments.

Tinnitus, the perception of sound in the absence of an external sound source, often develops after exposure to loud sounds (Hoffman and Reed, 2004; Møller, 2011; Baguley et al., 2013). In response to the cochlear damage by sound, the central auditory system increases its gain to compensate for the reduced sensorineural input from the cochlea (Salvi et al., 2000; Schaette and McAlpine, 2011; Galazyuk et al., 2012; Auerbach et al., 2014). The result of the gain change is the development of hyperactivity or elevated spontaneous firing in both the auditory system and non-auditory brain structures. This hyperactivity has been proposed as an underlying mechanism in tinnitus (Gerken, 1996; Salvi et al., 2000; Eggermont and Roberts, 2004; Roberts et al., 2010; Galazyuk et al., 2012; Salloum et al., 2016; Shore et al., 2016). Hyperactivity has been reported in the cochlear nucleus (Kaltenbach and Afman, 2000; Brozoski and Bauer, 2005), medial geniculate nucleus (Kalappa et al., 2014) and auditory cortex (Robertson and Irvine, 1989; Syka and Rybalko, 2000; Noreña et al., 2003). For the inferior colliculus, however, results from different studies are contradictory (for review see Shore and Wu, 2019). Some studies show hyperactivity or increased spontaneous activity caused by sound exposure (Ma et al., 2006; Mulders and Robertson, 2013; Berger et al., 2014; Longenecker and Galazyuk, 2016), whereas other studies found firing activity of IC neurons of sound-exposed animals to be no different from control animals (Heeringa and van Dijk, 2014; Shaheen and Liberman, 2018). There is another gap in our knowledge about sound trauma-induced changes in the IC. Even if hyperactivity occurs in IC, we do not know if it is created internally or is inherited from some auditory structures that provided inputs to the IC. According to current literature, the cellular mechanisms underlying tinnitus-linked hyperactivity in the cochlear nucleus and the thalamocortical circuit are distinct. In the cochlea nucleus, potassium and HCN channels were found to be important for tinnitus generation and resilience (Pilati et al., 2012a,b; Li et al., 2013, 2015). However, hyperactivity in the auditory cortex (AC) has been shown to arise from reduced inhibition within cortical circuits caused by decreased input to the AC (Yang et al., 2011; Llano et al., 2012).

Recently, our research revealed that unilateral sound exposure induces hyperactivity, with the most pronounced effect observed in the ipsilateral IC, but not in the contralateral IC as was expected (Longenecker and Galazyuk, 2016; Hsiao and Galazyuk, 2021). The main goal of the present study was to determine the source of hyperactivity in the IC, specifically investigating whether it is driven by presynaptic input or originates internally, while also exploring the underlying mechanism involved. Confirming results of our previous studies, we found that unilateral sound exposure caused hyperactivity in IC neurons with the most robust effect in the ipsilateral IC. We also showed that hyperactivity in the IC partially arises from the depolarization of neuronal resting membrane potentials (RMPs) following sound exposure. Further, for the first time our study demonstrated that in control (unexposed) mice, there exists a gradient of resting membrane potentials in the IC, whereby IC neurons exhibit more hyperpolarized RMPs with increasing depth or corresponding to the neuron’s best frequency (BF). Furthermore, this gradient was altered in the exposed animals, with a more pronounced effect observed in the ipsilateral IC.

## Materials and methods

### Subjects

A total of 24 CBA/CaJ mice were included in this study, with 16 mice allocated to the control group and 8 mice to the sound exposure (SE) group. The age of all animals ranged between 5 and 12 months. Mice were housed in pairs in a colony room with a 12 h light–dark cycle at a temperature of 25°C. The animal procedures conducted in this study were approved by the Institutional Animal Care and Use Committee at Northeast Ohio Medical University.

### Sound exposure

**Figure 1:**
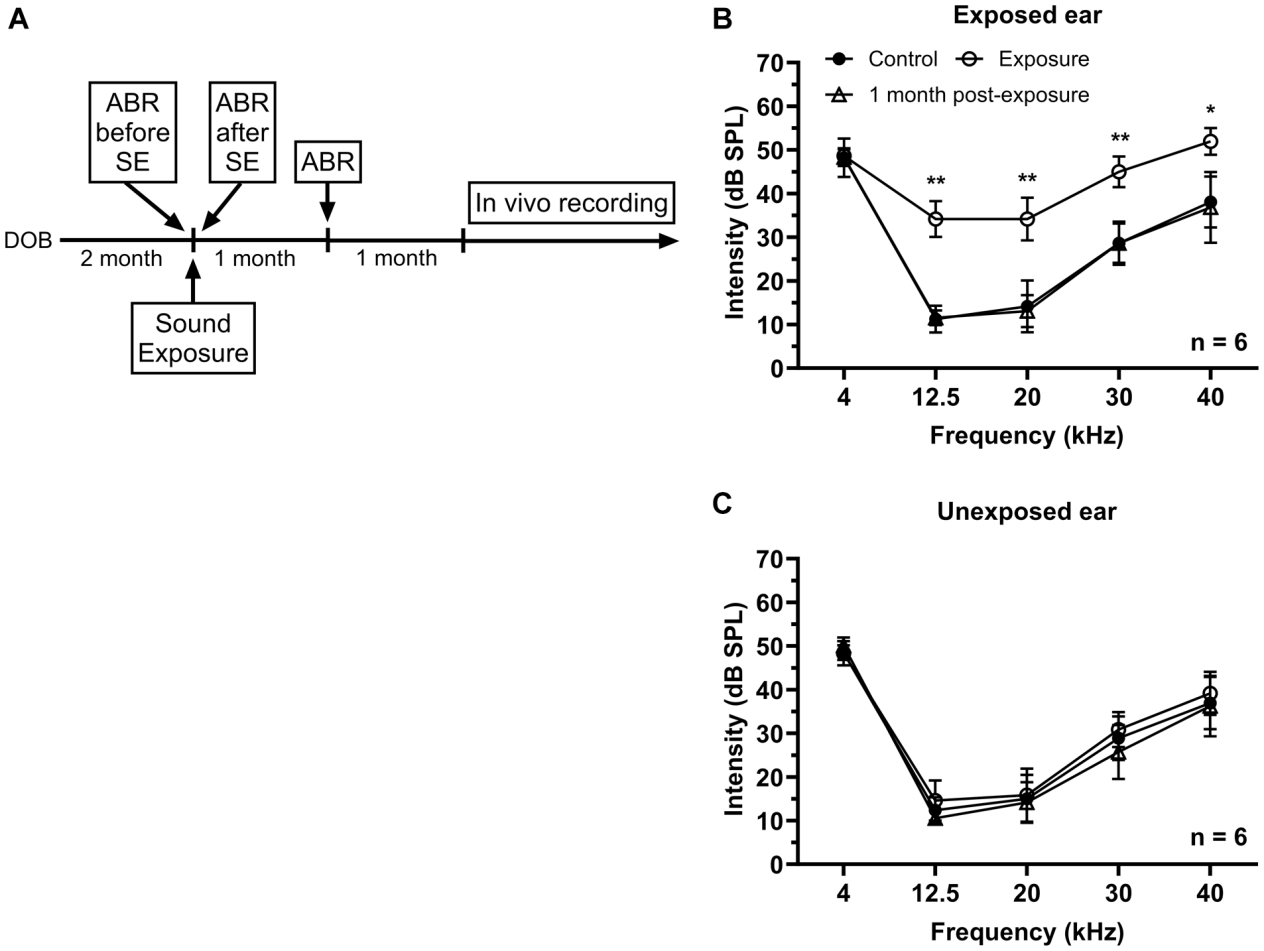
Effects of unilateral acoustic trauma on ABR threshold. **(A)** The timeline of experimental procedures. **(B)** ABR thresholds in the exposed ear were temporarily elevated and then returned to the control level one month post-exposure. **(C)** Sound exposure has no significant effect on ABR thresholds in the unexposed (blocked) ear. DOB, day of birth. *p < 0.05, **p < 0.01.

The animals used in the study were at least 2 months old at the time of sound exposure (Figure 1A). The sound exposure procedure was performed under general anesthesia, with a ketamine/xylazine mixture (100/10 mg/kg) *via* intraperitoneal injection. Additional intramuscular injections of 50% the initial dose was given to maintain the desired level of anesthesia. Unilateral sound exposure was performed by presenting a one-octave narrowband noise centered at 12.5 kHz (8–17 kHz) to the mice for 1 h. The noise was generated using a wave form generator (Wavetek model 395), amplified (Sherwood RX-4109) to 116 dB Sound Pressure Level (SPL), and played through an open field loudspeaker (Fostex FT17H) in a soundproof chamber. The open field loudspeaker was calibrated using a 0.25-inch microphone (Type 4944-B, Brüel and Kjaer). Before sound exposure, the left external ear canal of exposed mice was blocked with a foam earplug (3 M classic earplugs, 3 M company), followed by a Kwik-Sil silicone elastomer plug (World Precision Instruments). This manipulation typically reduces sound level by 30–50 dB SPL (Turner et al., 2006; Ropp et al., 2014).

### Auditory brainstem responses

Mice were anesthetized with ketamine/xylazine (100 and 10 mg/kg, respectively). Auditory brainstem responses were recorded in response to 5 ms tone bursts (0.5 ms rise/fall time) presented at frequencies of 4, 12.5, 20, 30, and 40 kHz with the sound level ranged from 80 to 10 dB SPL in 10 dB steps using an RZ6 multiI/O processor (Tucker-Davis Technologies). Tone bursts were delivered at the rate of 50/s through a speaker (LCY K-100 Ribbon Tweeter, Madisound), which was placed 10 cm in front of the animal’s head. ABR thresholds were measured before, directly following, and 1 month after sound exposure (Figure 1A). Stainlesssteel electrodes (disposable subdermal needle electrode, LifeSync Neuro) were placed subdermally at the vertex (active), the ipsilateral and contralateral mastoids (references), and at the base of animal’s tail (ground). The evoked potentials were amplified (RA4PA MEDUSA Preamp, Tucker-Davis Technologies), filtered (100–3,000 Hz bandpass), and averaged across 300 repetitions. Thresholds were determined by visual examination of the averaged ABR waveforms in response to each frequency and sound level combination.

### Surgery

Each mouse was anesthetized with 1.5–2.0% isoflurane during surgery. The hair covering the skull surface was removed by using depilatory lotion. A midline incision was made and the tissue over the cranium was removed. A small metal rod was then attached to the cranium using dental cement (C&B Metabond, Japan). After the surgery, antibiotic ointment was applied to the surgical areas, and the animal was returned to the holding cage to observe its recovery from anesthesia for at least 2 h. Following a recovery period of at least 2 days, each animal was trained to stay in a holding device in a single-walled sound attenuating booth. The holding device consisted of a custom-made small plastic tube and a small metal holder. During electrophysiological recordings, animals’ ears were unobstructed to allow for free-field acoustic stimulation.

### Acoustic stimulation

The stimulation protocol consisted of two sections. First, a 15 s silence recording window (no sound presented) was used to measure the spontaneous firing rate (SFR). Second, tone pips with a duration of 100 ms were presented at a wide range of sound frequencies (3–51 kHz, 2 kHz step) at the level of 55 dB SPL to assess best frequency (BF) for each recorded neuron. The tone pips were generated by a Tucker-Davis Technologies system 3, which included an RX6 multifunction processor, a PA5 programmable attenuator, and the SigGenRP software. The tone pips were then amplified using an amplifier (HCA-750A, PARASOUND) and delivered to the animal *via* a free-field loudspeaker (LCYK100 Ribbon Tweeter, Madisound). The loudspeaker was positioned 10 cm in front of the animal at a 25° angle into the sound field contralateral to the IC. The sound stimuli were calibrated using a 1/4-inch free-field microphone (Type 4939, Brüel and Kjær) positioned 10 cm in front of the speaker. A conditioning amplifier (NEXUS 2690-A, Brüel and Kjær) was employed at a sampling rate of 195.3 kHz to ensure precise measurement of sound pressure levels across different frequencies.

### Intracellular recording

Intracellular recordings were performed using quartz microelectrodes (1.0-mm-diam with filament, Sutter Instruments, Novato, CA) filled with 1 M potassium acetate. The electrodes were pulled using a Flaming-Brown micropipette puller (P2000, Sutter Instrument) and had impedances ranging from 100 and 300 MΩ. The electrode was advanced into the IC in 2-μm steps using a precision microdrive (Model 660, KOPF Instrument). The intracellular responses of IC neurons were amplified (Model IR183A, Cygnus Technology) and monitored on a digital oscilloscope (DLM 3024, Yokogawa). The waveforms were digitized at a sampling rate of 100 kHz by a data acquisition system (EPC-10, Heka) and stored on a computer hard drive.

Recordings were conducted from both the ipsilateral and contralateral IC relative to the side of sound exposure in unanesthetized mice inside a single-walled sound attenuating booth (Industrial Acoustics Company, Inc.). Throughout the 3–5 h recording session, the animal was periodically offered water and monitored for any signs of discomfort. After each recording session, the exposed skull was covered with a Kwik-Sil silicone elastomer plug (World Precision Instruments) and the animal was returned to its holding cage. Experiments were conducted at least 2 months post-exposure in the SE group and recordings were performed every other day for up to 2 weeks. At the end of the experiment, the animal was sacrificed with an IP injection of Fatal-Plus. No sedative drugs were used during the recording sessions. If the animal showed any signs of discomfort, the recording session was terminated, and the mouse was returned to its cage.

### Data analysis

For analysis of ABR data, we used a one-way ANOVA along with a Dunn’s post-hoc test to compare the thresholds across varying frequencies observed within the three different experimental time points. For each neuron, we assessed several parameters including the spontaneous firing rate (SFR), resting membrane potential (RMP), best frequency (BF), and half width of action potentials. The RMP was determined by analyzing the averaged waveforms over their entire duration of recording. First, we calculated the mean value of the averaged waveform, then excluded any values that deviated beyond one standard deviation (SD) from the mean, and finally recalculated a new mean. This method for calculating resting potential allowed us to accurately determine the RMP while minimizing the influence of spikes or large postsynaptic potentials. Sound stimulus-evoked potentials were defined as transient depolarizing or hyperpolarizing fluctuations from the RMP that surpassed 2 SD (95% confidence limits) and began after stimulus onset. Custom made software was used for spike data analysis.

All statistical analyses were accomplished using GraphPad Prism 9 (version 9.5.1., GraphPad). The Mann–Whitney test was used to compare the control and sound exposure groups. For multiple comparisons, a Kruskal-Wallis test followed by Dunn’s post-hoc test was applied. Data are presented as mean with either the standard deviation (SD) or the standard error of the mean (SEM). A significance level of *p* < 0.05 was used to determine statistical significance.

Due to limitations in the number of some measurements, we corrected for the bias associated with small samples of the response probability distribution by using the method of Panzeri and Treves (1996). To obtain a measure of 95% confidence intervals a bootstrap method was implemented. Input data matrices were generated by sampling, at random and with replacement from the original data matrix. The number of bootstrap samples for a given measurement category was based on the level of an expected sample size. The mean and variance at a size of not more than 100 resampled points was used for each of the bootstrapped measurements. The mean and 95% confidence intervals were then obtained for each measure from this distribution.

## Results

In this study two groups of mice were used – control (*n* = 16) and sound exposed (*n* = 8). The sound exposed group was exposed to a narrowband noise centered at 12.5 kHz (8–17 kHz) presented at 116 dB SPL during one hour under ketamine/xylazine anesthesia at the age of 2–3 months. ABRs were collected before and right after sound exposure to make sure that the exposure was effective in causing a significant temporally threshold shift. Then animals were allowed to recover from acoustic trauma for one month followed by another ABR recording (Figure 1A). After sound exposure, a transient elevation in threshold was observed at the frequencies of 12.5, 20, 30, and 40 kHz in the affected ear, which subsequently returned to the control levels after one month (Figure 1B). In contrast, ABR thresholds in the unexposed (blocked) ear remained unaffected by the sound exposure (Figure 1C).

### The spontaneous firing rate of IC neurons was increased after unilateral sound exposure

**Figure 2:**
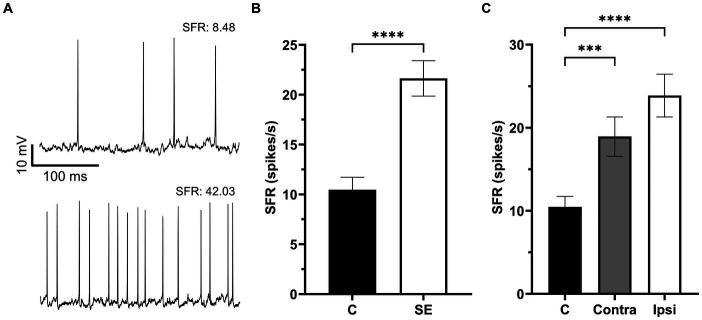
The Spontaneous firing rate was increased in IC neurons after sound exposure. **(A)** Representative recording traces of two neurons with low and high SFR. **(B)** The SFR in the control (*n* = 132, mean = 10.47 ± 1.26, median = 5.07) and SE groups (*n* = 170, mean = 21.63 ± 1.77, median = 13.35). **(C)** The SFR in IC neurons of the control (*n* = 132, mean = 10.47 ± 1.26, median = 5.07), Contra- (*n* = 77, mean = 18.94 ± 2.37, median = 10.18) and Ipsi-groups (*n* = 93, mean = 23.87 ± 2.57, median = 14.5). ***p < 0.001, **** *p* < 0.0001.

A total of 302 IC neurons were recorded and their SFR were assessed in control and sound exposure groups. The average SFRs of IC neurons in the control group was 10.47 ± 1.26 spikes/s and it was no different between the right and left ICs (right, 8.0 ± 1.24 spikes/s; left, 9.7 ± 1.85 spikes/s, *p* = 0.88). We found a significant increase in SFR two months after sound exposure (Figures 2A,B). Then we divided the sound exposure (SE) group into contralateral and ipsilateral IC neurons relative to the side of sound exposure. Both contralateral and ipsilateral IC neurons exhibited a significant increase in SFR compared to the control group, with the increase being more pronounced in the ipsilateral IC (Figure 2C).

### The RMP in IC neurons was no different after sound exposure

**Figure 3:**
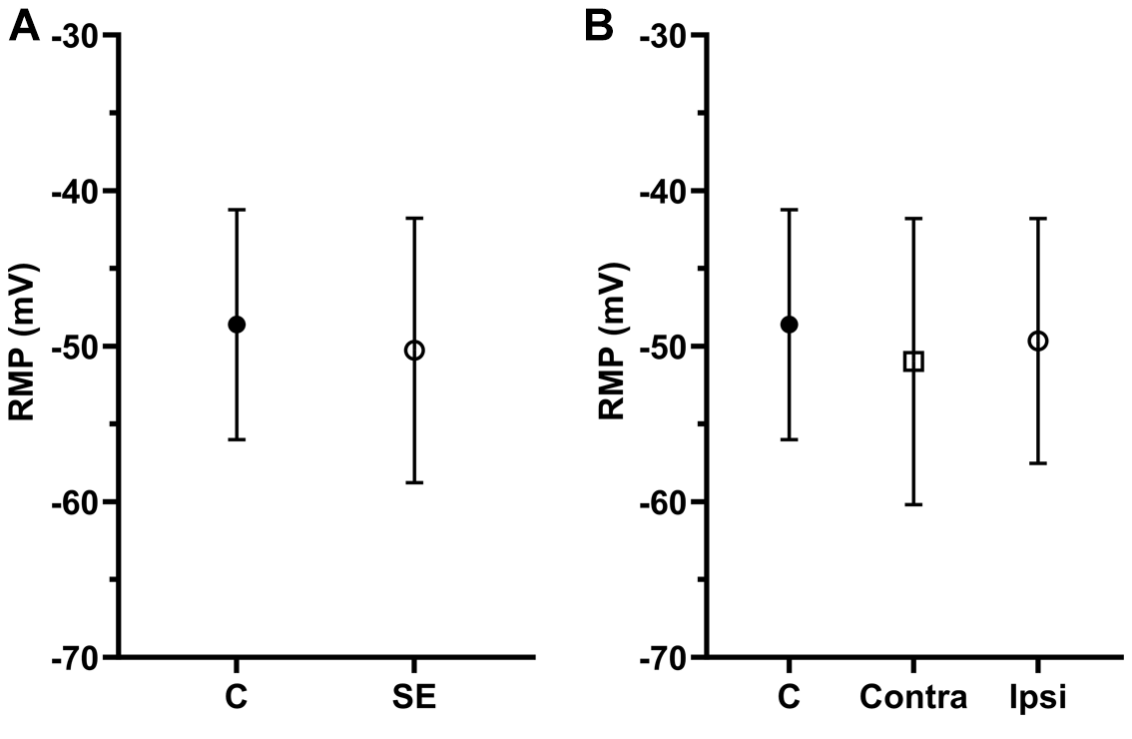
The mean of RMPs in IC neurons was not significantly affected by sound exposure. **(A)** The mean RMP in the control (*n* = 117, mean = −48.61 ± 7.39) and SE groups (*n* = 164, mean = −50.27 ± 8.5). **(B)** The mean RMP in the control (*n* = 117, mean = −48.61 ± 7.39), exposed contralateral (*n* = 75, mean = −50.99 ± 9.2) and exposed ipsilateral groups (*n* = 89, mean = −49.66 ± 7.87).

Resting membrane potentials were evaluated in 281 IC neurons, with RMPs ranging from-40.19 to −73.83 mV. The average RMPs of IC neurons in the control group was −48.61 ± 7.39 mV and it was no different between the right and left ICs (right, −47.42 ± 7.64 mV; left, −48.84 ± 8.01 mV, *p* = 0.22). We found no significant difference in RMPs between the control and SE group (Figure 3A). Similar to analysis of SFR, we divided all neurons in the SE group into contra- and ipsilateral groups related to the side of sound exposure. Comparison of RMPs between these two groups revealed no significant difference (Figure 3B).

### IC neurons with BFs at or above center frequency of exposure become depolarized after sound exposure

**Figure 4:**
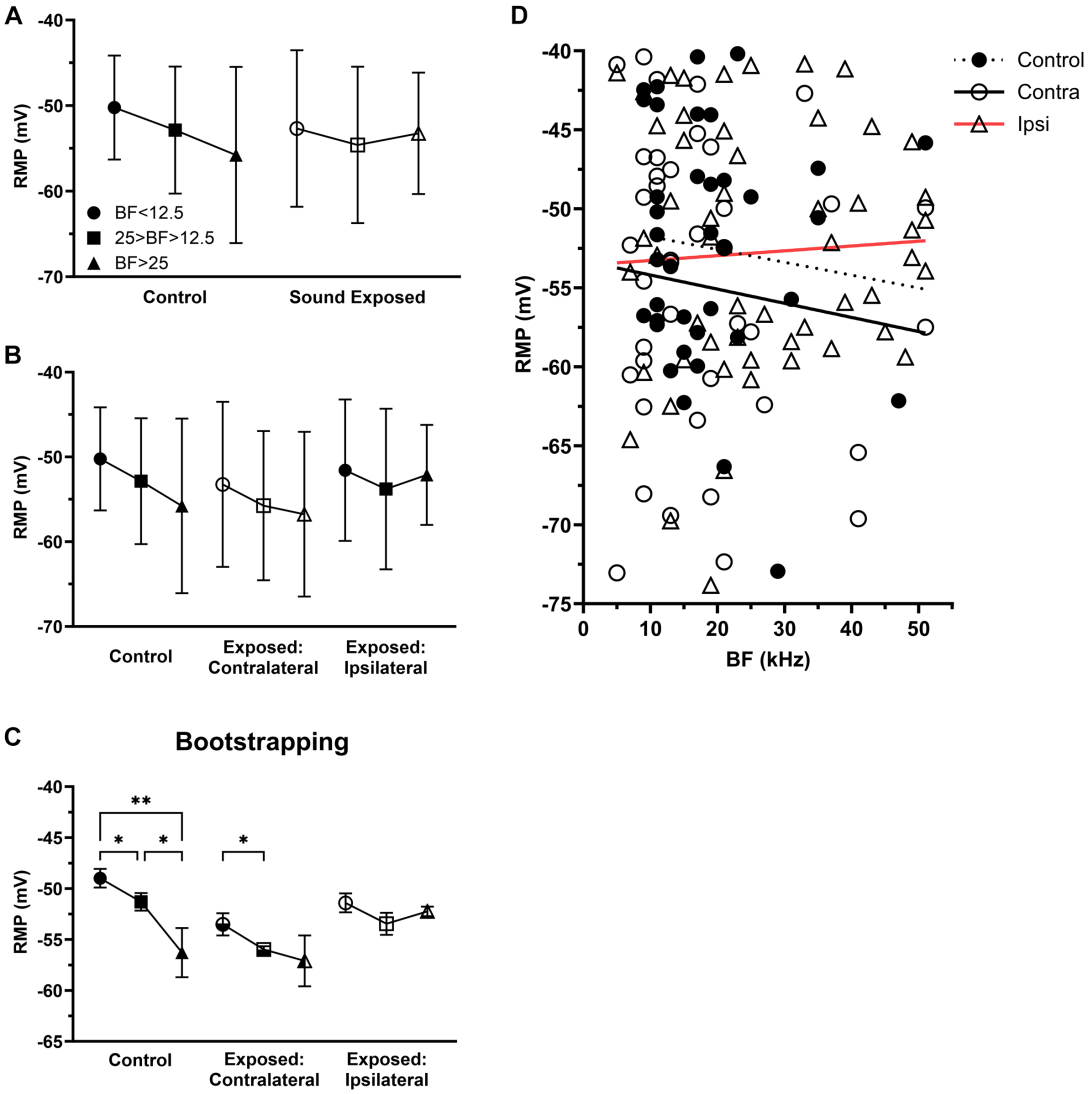
After sound exposure RMPs were depolarized with BFs at or above center frequency of exposure. **(A)** The RMP in the three BFs ranges of control (BF < 12.5, *n* = 12, mean = −50.23 ± 6.08; 25 > BF > 12.5, *n* = 20, mean = −52.86 ± 7.42; BF > 25, *n* = 6, mean = −55.78 ± 10.3) and SE group (BF < 12.5, *n* = 24, mean = −52.68 ± 9.15; 25 > BF > 12.5, *n* = 41, mean = −54.59 ± 9.14; BF > 25, *n* = 29, mean = −53.24 ± 7.1). **(B)** The RMP in the three BFs ranges of control, contralateral (BF < 12.5, *n* = 16, mean = −53.23 ± 9.74; 25 > BF > 12.5, *n* = 17, mean = −55.74 ± 8.8; BF > 25, *n* = 7, mean = −56.75 ± 9.72) and ipsilateral group (BF < 12.5, *n* = 8, mean = −51.56 ± 8.33; 25 > BF > 12.5, *n* = 24, mean = −53.78 ± 9.47; BF > 25, *n* = 22, mean = −52.12 ± 5.9). **(C)** Bootstrapping approach supports hypothesis that sound exposure alters RMP gradient in both contralateral and ipsilateral ICs predominantly at and above the frequency range of exposure. **(D)** The relationship between RMP and BF within the population of IC neurons across the control (*n* = 38, *R*^2^ = 0.0114), contralateral (*n* = 78, *R*^2^ = 0.0135), and ipsilateral groups (*n* = 132, *R*^2^ = 0.0028). *p < 0.05, **p < 0.01.

Our recent study has shown that the sound exposure effect is strongest in the ipsilateral ICs and the most affected neurons by sound exposure have characteristic frequencies at or above center frequency of exposure (Hsiao and Galazyuk, 2021). If so, it is possible that the most robust changes after sound exposure would be expected in the ipsilateral IC, where neurons are tuned to frequencies at and above the center frequency of sound exposure. To test this hypothesis, we divided all IC neurons into three frequency ranges based on their BFs. The first group consisted of neurons with BFs below 12.5 kHz (the center frequency of sound exposure). The second group included neurons with BFs ranging from 12.5 to 25 kHz (one octave above the center frequency of sound exposure) and the third group comprised neurons with BFs above 25 kHz (two octaves above center frequency of sound exposure) (Figures 4A–C).

Although statistically not significant, we observed a trend for RMP gradient in IC neurons in the control group, where their RMPs tended to hyperpolarize with their BFs (Figure 4A). In the SE group, this trend was change in neurons with BFs above 25 kHz (Figure 4A). Further, dividing all SE neurons into contralateral (Figures 4B,D) and ipsilateral (Figures 4B,D) groups revealed that the most affected neurons were in the ipsilateral IC neurons with BFs above 25 kHz.

We hypothesize that the lack of significance in our findings was primarily due to the small sample size of our dataset. To test this hypothesis, we applied the bootstrapping approach to increase the sample size. This approach allowed us to demonstrate the statistical significance of both the trend observed in the control animals and the effect on this trend in the ipsilateral IC (Figure 4C).

### The half width of action potentials was decreased after sound exposure

**Figure 5:**
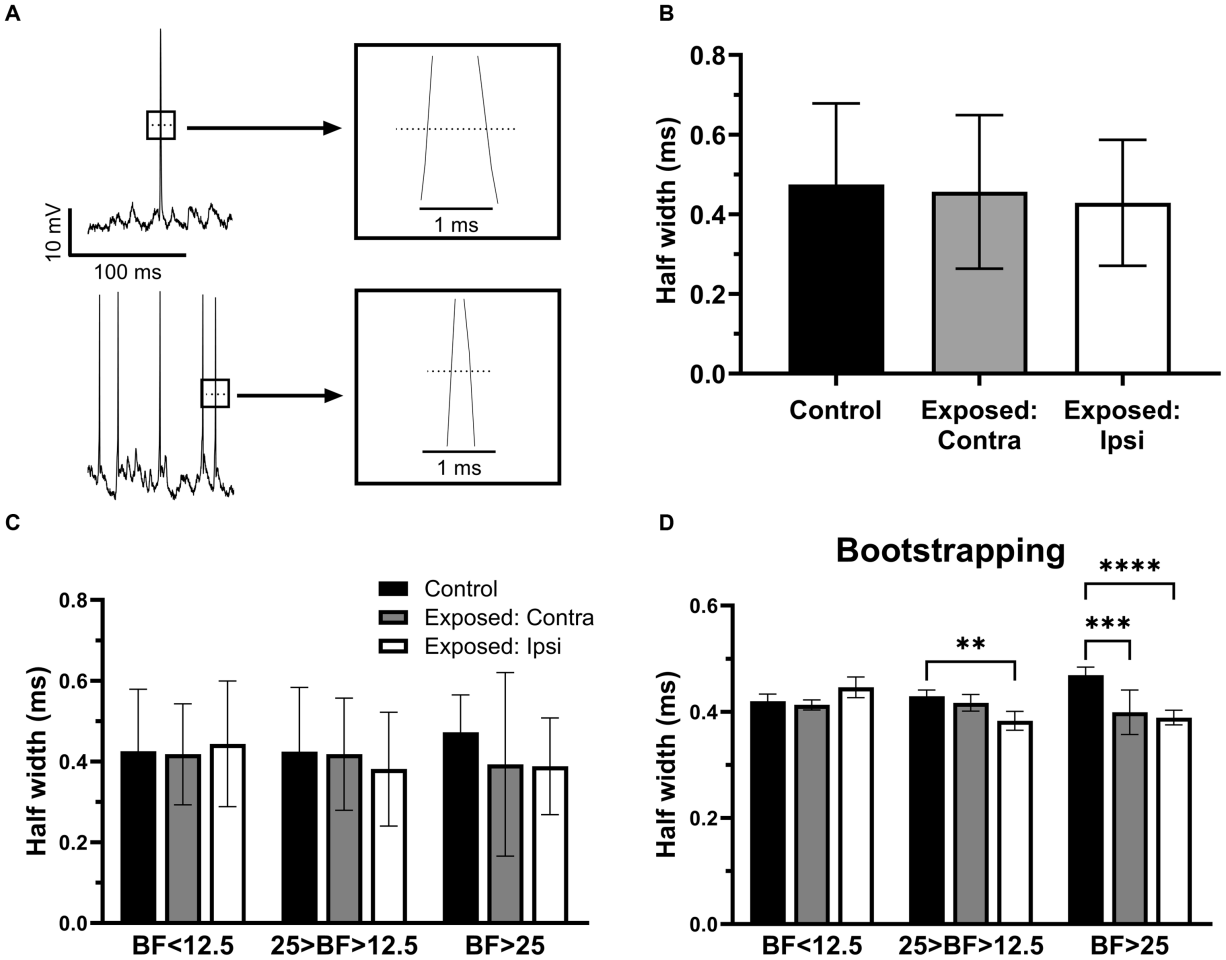
The half width of action potentials was decreased after sound exposure. **(A)** Representative recording traces from two neurons with wide and narrow half width of action potentials. **(B)** The half width of action potentials in control (*n* = 145, mean = 0.47 ± 0.2), contra (*n* = 74, mean = 0.46 ± 0.19) and ipsilateral groups (*n* = 103, mean = 0.43 ± 0.16). **(C)** The half width of action potentials in the three BFs ranges of control (BF < 12.5, *n* = 13, mean = 0.43 ± 0.15; 25 > BF > 12.5, *n* = 17, mean = 0.42 ± 0.16; BF > 25, *n* = 8, mean = 0.47 ± 0.09), contralateral IC (BF < 12.5, *n* = 14, mean = 0.42 ± 0.13; 25 > BF > 12.5, *n* = 16, mean = 0.42 ± 0.14; BF > 25, *n* = 7, mean = 0.39 ± 0.23) and ipsilateral ICs (BF < 12.5, *n* = 11, mean = 0.44 ± 0.16; 25 > BF > 12.5, *n* = 17, mean = 0.38 ± 0.14; BF > 25, *n* = 22, mean = 0.39 ± 0.12). **(D)** Bootstrapping approach supports hypothesis that sound exposure reduces half width of action potentials predominantly affecting ipsilateral IC at and above the frequency range of exposure. **p < 0.01, ***p < 0.001, **** *p* < 0.0001.

To further investigate whether sound exposure affected intrinsic properties of IC neurons, we measure and compared the half width of action potentials (Figure 5A) between the control and exposed groups. We found that the half width of action potentials was decreased after sound exposure (Figure 5B). Consistent with the RMP analysis, both the contralateral (Figure 5C) and ipsilateral IC (Figure 5C) exhibited a decrease in the half width of action potentials compared to the control group (Figure 5C). Notably, this decrement in half width of action potentials was more pronounced in IC neurons with BFs above 25 kHz (Figure 5C). Bootstrapping of these data supported our hypothesis and shows a statistical significance in the effect for the neurons with BFs ranging from 12.5 kHz to 25 kHz in the ipsilateral IC. At the BFs above 25 kHz, both contralateral and ipsilateral ICs exhibited a significant decrease in the half width of action potentials following sound exposure (Figure 5D).

### The proportion of sound evoked response types was not affected by sound exposure

**Figure 6:**
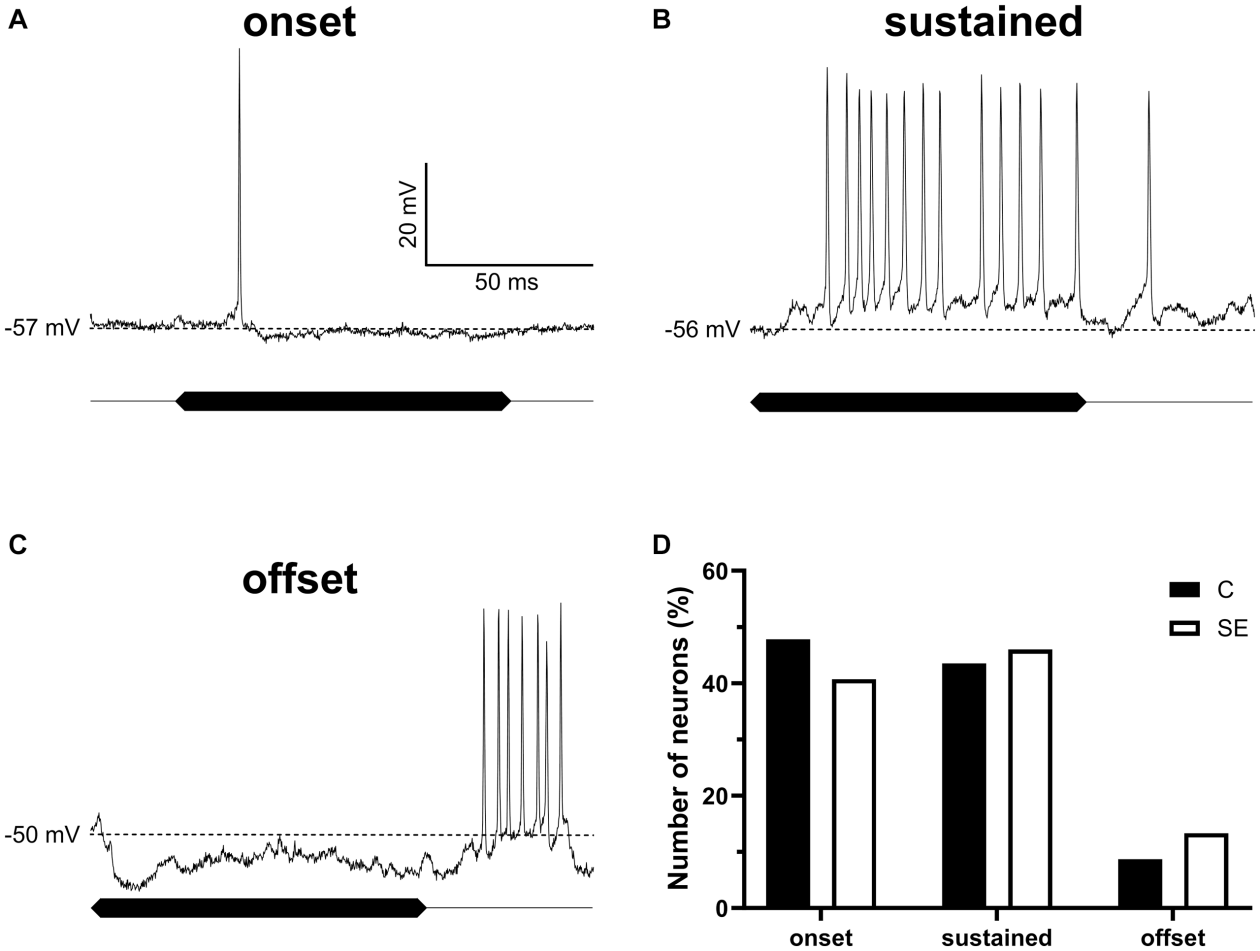
The proportion of IC neurons with the three sound evoked response types was not significantly affected by sound exposure. **(A–C)** Three representative IC neurons showing onset, sustained and offset response types. **(D)** The proportion of onset (control, 47.8%, *n* = 22; SE, 40.7%, *n* = 46), sustained (control, 43.5%, *n* = 20; SE, 46%, *n* = 52) and offset (control, 8.7%, *n* = 4; SE, 13.3%, *n* = 15) response types in control and SE groups. The dash line indicates RMP values of which are labeled on the left of each trace.

There is a possibility that sound exposure might affect basic response properties of IC neurons to sound. To test for this possibility, we assessed and compared response properties of IC neurons in control and sound exposed groups. For this comparison all neurons were divided into three basic response types (onset, sustained, and offset) based on their responses to 100 ms duration pure tones presented at neuron’s BF at the sound level of 55 dB SPL. Intracellular recordings of the three representative neurons with these three response types are shown in Figures 6A–C. The total of 159 neurons in control and SE group contributed to our data analysis. In both groups, IC neurons with onset (Figure 6D) and sustained (Figure 6D) response types dominated. The offset response type, however, was observed in much smaller population of IC neurons in both control and SE groups (Figure 6D). Thus, we did not observe significant changes in the proportion of the three response types after sound exposure.

## Discussion

The present study made an important step forward toward our understanding of hyperactivity development after sound exposure. It identified the target for future *in-vitro* studies – IC region (ipsilateral, at and above the frequency range of exposure) and the postsynaptic change (depolarization of RMPs). Sharp electrode recording technique allowed us to sample a large population of neurons (*n* = 302) in unanesthetized animals with minimal disruption of brain circuits and neuronal intracellular environment. However, it was not capable of determining neuronal ionic mechanism due to higher input resistance and typically shot recording time. The patch clamp technique, however, was designed to assess ionic cellular mechanisms due to very low access resistance, but impractical of screening a large population of neurons in live animals. Therefore, future *in-vitro* studies should be used to determine cellular mechanism(s) underlying the sound exposure-induced RMP depolarization in the IC.

This study reveals four primary findings: (1) unilateral sound exposure leads to an increase in spontaneous activity among IC neurons, with the most pronounced effect observed in the ipsilateral IC (Figure 2); (2) in exposed animals, IC neurons with BFs at and above the center frequency of sound exposure show more depolarized RMPs, especially in the ipsilateral IC (Figure 4); (3) in the control (unexposed) mice, there is a distinct RMP gradient where RMPs of IC neurons are more hyperpolarized with depth or neuron’s BF. However, this gradient is altered in the exposed animals, predominantly in the ipsilateral IC (Figure 4); (4) the spike half width of the IC neurons is reduced in exposed animals (Figure 5). This discussion focuses on the novel and most significant of these findings.

### Unilateral sound exposure causes more pronounced hyperactivity in the ipsilateral IC

This study, along with two more research publications from our laboratory (Longenecker and Galazyuk, 2016; Hsiao and Galazyuk, 2021) provides strong evidence that unilateral sound exposure induces hyperactivity or increased spontaneous firing activity in both the contralateral and ipsilateral IC. However, the most pronounced effect is observed in the ipsilateral IC. One possible explanation for this finding is that unilateral sound exposure leads to maladaptive changes in neuronal firing or hyperactivity in both the ipsilateral and contralateral cochlear nuclei, which subsequently project to both ICs. Previous studies have reported plastic changes in the contralateral cochlear nucleus in response to unilateral auditory deprivation, including identified alterations in glutamatergic synapses in both the affected and unaffected cochlear nuclei (Rubio, 2006; Whiting et al., 2009). However, the reason behind the stronger effect of unilateral sound exposure on the ipsilateral IC remains to be determined. Nonetheless, this finding is very important for the field of tinnitus research, as hyperactivity or increased spontaneous firing is widely recognized as being associated with tinnitus. Currently, hyperactivity has been demonstrated at all levels of the central auditory pathway. However, for the IC, consensus regarding tinnitus-related hyperactivity after sound exposure is lacking (for review see Shore and Wu, 2019). Some studies show hyperactivity or increased spontaneous activity caused by sound exposure (Ma et al., 2006; Mulders and Robertson, 2013; Berger et al., 2014; Longenecker and Galazyuk, 2016; Ma et al., 2020), while others find no significant difference compared to the control (Heeringa and van Dijk, 2014; Shaheen and Liberman, 2018). This discrepancy can be explained by the fact that the majority of these studies focus on the contralateral IC following unilateral sound exposure, where the hyperactivity is not as prominent as in the ipsilateral IC. Indeed, in the present study, neurons in the contralateral IC do show some hyperactivity, but it is relatively weak compared with the ipsilateral IC.

### The RMPs of the hyperactive IC neurons show depolarization

Our results showed that unilateral sound exposure induces depolarization of neuronal resting membrane potentials, particularly in the ipsilateral IC where more robust hyperactivity is observed (Figures 2, 4). Two potential mechanisms could account for this depolarization. First, it may be attributed to a release from tonic inhibition. In normal, unexposed animals, this inhibition could contribute to hyperpolarization of RMPs in IC neurons. However, after sound exposure, this inhibition might be reduced, leading to a depolarizing shift. In support of this hypothesis, a new type of GABAergic neurons that express neuropeptide Y has been recently discovered in the IC of the mouse (Silveira et al., 2020). These principal GABAergic neurons, comprising one-third of the inhibitory neurons in the IC, exhibit spontaneous firing and provide tonic inhibition to their postsynaptic targets. If the activity of these neurons is reduced by the sound exposure, the RMPs of their target neurons would likely to be depolarized due to a release from tonic inhibition. This study strongly advocates that the reduction of RMP in auditory neurons may be partially explained by a decrease in GABAergic contribution to RMPs after sound exposure. Indeed, down-regulation of inhibitory neurotransmission, related to partial peripheral deafferentation resulting from acoustic trauma, consistently underlies the neuronal hyperactivity observed in animal models of tinnitus (Caspary and Llano, 2017). Another mechanism that could account for RMP depolarization in our experiments is the alternation of potassium channels after sound exposure. These channels play a crucial role in setting the resting membrane potential and controlling the duration, shape and firing frequency of action potentials. Potassium channels are localized in all subcellular compartments critical for the electrical conduction of excitatory inputs. It has been shown that hyperactivity of fusiform cells in the dorsal cochlear nucleus is, at least in part, caused by decreased Kv7.2/3 (KCNQ2/3) potassium currents (Li et al., 2013, 2015).

### RMP gradient in the IC

Another novel finding in this study is an RMP gradient in the IC, where RMPs of IC neurons became more hyperpolarized with depth or with neuron’s BF (Figure 4). This gradient was altered by sound exposure. Consistent with sound exposure-induced hyperactivity, the changes in the gradient were most pronounced in the ipsilateral IC for neurons with BFs at and above the center frequency of sound exposure. Various gradients are not so unique to the IC. In addition to the well-established *CF* gradient with depth along the dorsolateral to ventromedial axis (Ehret, 1997; Schreiner and Langner, 1997; Casseday et al., 2002; Ehret and Schreiner, 2005), nine other spatial gradients have been identified (Ehret et al., 2005). These gradients relate to tone-response threshold (Stiebler and Ehret, 1985; Stiebler, 1986; Seshagiri and Delgutte, 2007), tone-response latency and latency jitter (Schreiner and Langner, 1988; Hattori and Suga, 1997; Langner et al., 2002; Straka et al., 2014), best-modulation frequency to amplitude modulated tones or so-called periodotopy (Schreiner and Langner, 1988; Langner et al., 2002; Baumann et al., 2011), sharpness of frequency tuning to excitatory tones (Schreiner and Langner, 1988), shapes of excitatory frequency response areas (Ehret et al., 2003; Hage and Ehret, 2003), temporal tone-response patterns (Hage and Ehret, 2003), and preferred responses to velocities and directions of frequency sweeps (Hage and Ehret, 2003). Furthermore, immunohistochemistry sections of rat and mice IC reveal a gradient of GABAergic and glycinergic inputs from ventrolateral to dorsomedial along the axis with GABAergic inputs dominated in the dorsomedial IC (Choy et al., 2015). The systematic distributions of various neuronal response properties along specific coordinates in the three-dimensional IC space provide insights into the organization of response diversities and their potential functional relationships with auditory perceptual abilities and acuities.

## Data availability statement

The raw data supporting the conclusions of this article will be made available by the authors, without undue reservation.

## Ethics statement

The animal study was approved by Institutional Animal Care and Use Committee at Northeast Ohio Medical University. The study was conducted in accordance with the local legislation and institutional requirements.

## Author contributions

C-JH: Data curation, Formal analysis, Investigation, Visualization, Writing – original draft, Writing – review & editing. AG: Data curation, Formal analysis, Investigation, Visualization, Writing – original draft, Writing – review & editing, Conceptualization, Funding acquisition, Methodology, Project administration, Resources, Supervision, Validation.

## Funding

The author(s) declare financial support was received for the research, authorship, and/or publication of this article. This research was supported by grant R01 DC016918 from the National Institute on Deafness and Other Communication Disorders of the U.S. Public Health Service.

## Conflict of interest

The authors declare that the research was conducted in the absence of any commercial or financial relationships that could be construed as a potential conflict of interest.

## Publisher’s note

All claims expressed in this article are solely those of the authors and do not necessarily represent those of their affiliated organizations, or those of the publisher, the editors and the reviewers. Any product that may be evaluated in this article, or claim that may be made by its manufacturer, is not guaranteed or endorsed by the publisher.
